# Treatment restrictions after acute stroke across nine European countries and their association with 90-day mortality: a post hoc analysis of the PRECIOUS randomised controlled trial

**DOI:** 10.1016/j.lanepe.2026.101894

**Published:** 2026-10-14

**Authors:** Wouter M. Sluis, Wouter A.C. van Amsterdam, Jeroen C. de Jonge, Hendrik Reinink, George Ntaios, Janika Kõrv, Anne Hege Aamodt, Götz Thomalla, Laszlo Csiba, Alfonso Ciccone, Iwona Kurkowska-Jastrzebska, Malcolm R. Macleod, Philip M. Bath, H Bart van der Worp, PRECIOUS investigators, Hendrik Reinink, Jeroen C. de Jonge, H. Bart van der Worp, Philip M. Bath, Diederik van de Beek, Eivind Berge, Alfonso Ciccone, Laszlo Csiba, Jacques Demotes, Diederik W. Dippel, Janika Korv, Iwona Kurkowska-Jastrzebska, Kennedy R. Lees, Malcolm R. Macleod, George Ntaios, Gary Randall, Götz Thomalla

**Affiliations:** a Department of Neurology and Neurosurgery, Brain Center, University Medical Center Utrecht, Heidelberglaan 100, 3584 CX, Utrecht, the Netherlands; b Julius Center for Health Sciences and Primary Care, University Medical Center Utrecht, Heidelberglaan 100, 3584 CX, Utrecht, the Netherlands; c Department of Neurology, St. Antonius Ziekenhuis, Soestwetering 1, 3543 AZ, Utrecht, the Netherlands; d Department of Neurology, Spaarne Gasthuis, Boerhaavelaan 22, 2035 RC, Haarlem, the Netherlands; e First Propaedeutic Department of Internal Medicine, AHEPA Hospital, Aristoteles University of Thessaloniki, Greece; f Department of Neurology and Neurosurgery, University of Tartu, L. Puusepa 8, 50406, Tartu, Estonia; g Department of Neurology, Oslo University Hospital, Sognsvannsveien 20, 0372, Oslo, Norway; h Department of Neurology, University Medical Center Hamburg-Eppendorf, Martinistraße 52, 20246, Hamburg, Germany; i Department of Neurology, University of Debrecen, Nagyerdei krt. 98, 4032, Debrecen, Hungary; j Department of Neurology and Stroke Unit, ASST di Mantova, Strada Lago Paiolo 10, 46100, Mantova, Italy; k Department of Neurology and Stroke Unit, ASST Papa Giovanni XXIII, Piazza OMS 1, 24127, Bergamo, Italy; l 2nd Department of Neurology, Institute of Psychiatry and Neurology, Sobieskiego 9, 02-957, Warsaw, Poland; m Centre for Clinical Brain Sciences, University of Edinburgh, Chancellor's Building, 49 Little France Crescent, EH16 4SB, Edinburgh, United Kingdom; n Stroke Trials Unit, Mental Health & Clinical Neuroscience, University of Nottingham, C Floor, South Block, Queen's Medical Centre, Derby Road, NG7 2UH, Nottingham, United Kingdom

**Keywords:** Intracerebral haemorrhage, Ischaemic stroke, Treatment restrictions, Do-not-resuscitate, DNR

## Abstract

**Background:**

In patients with acute stroke, early withholding or withdrawal of life-sustaining therapies is associated with an increased risk of death. We used practice variation across nine European countries to assess whether this relation may be causal.

**Methods:**

We performed a post hoc analysis of the PREvention of Complications to Improve OUtcome in elderly patients with acute Stroke (PRECIOUS) trial, a randomised clinical trial of metoclopramide, paracetamol, and/or ceftriaxone started within 24 h of symptom onset in older patients with acute ischaemic stroke or intracerebral haemorrhage (ISRCTN82217627). The frequency of early treatment restrictions, defined as those installed within 24 h after randomisation, was calculated per participating country and their relation with the primary outcome ‘death at 90 days’ was estimated through: 1) adjustment for confounders with regression analysis with inverse-propensity score reweighting; and 2) comparing patients enrolled in countries with and without early restrictions, adjusted for confounders with regression analysis.

**Findings:**

Of 1493 patients enrolled in PRECIOUS between 2016 and 2022, 1451/1493 (97%) were included in this analysis, of whom 276/1451 (19%) had an early treatment restriction. The rate of these restrictions ranged from 0% to 48% across the participating countries. Patients with restrictions were more likely to die within 90 days (inverse propensity-weighted odds ratio (OR) 3.39; 95% CI 2.71–4.25). However, there was no difference in the rate of death at 90 days between countries with and those without restrictions (21.3% vs 23.5%, adjusted OR 1.39; 95% CI 0.8–2.39).

**Interpretation:**

The use of treatment restrictions early after stroke varied widely across Europe. Although restrictions were independently associated with an increased rate of death, the comparison of death rates between countries with and those without such restrictions provides no indication that this relation is causal.

**Funding:**

European Union's Horizon 2020 research and innovation programme; grant agreement No. 634809.

**Research in context**

**Evidence before this study**

We searched PubMed from database inception to 28 February 2026 using combinations of the terms “stroke”, “ischaemic stroke”, “intracerebral haemorrhage”, “treatment restrictions”, “treatment limitations”, “withholding treatment”, “withdrawal of care”, “life-sustaining treatment”, “do-not-resuscitate”, “goals of care”, and “advance care planning.” Studies were eligible irrespective of whether these reported on the relation between such restrictions and outcomes. This search yielded four European (two from the Netherlands, one from the United Kingdom and one from Finland), seven Northern American (six from the United States and one from Canada), and two Asian (one from China and one from Taiwan) retrospective cohort studies.

The rate of treatment restrictions within these studies varied from 8 through 36% with treatment restrictions more frequently installed in European (34–36%) and Northern American (13–35%) than in Asian (8–12%) countries. Seven of these studies assessed the relation between treatment restrictions and outcomes, and all of these found an independent association between treatment restrictions and death or poor functional outcome. Due to their retrospective design, the studies had a high risk of bias and residual confounding is likely. Based on these findings, European guidelines for ICH suggest not implementing general policies involving do not attempt cardiopulmonary resuscitation (DNR) orders within the first 24 h after ICH onset, and American guidelines for ICH find aggressive care including postponement of new DNR orders until at least the secund full day of hospitalisation reasonable to decrease mortality and improve functional outcome.

**Added value of this study**

We combined the advances of prospectively collected data in the European PRECIOUS trial with analyses to limit residual confounding as much as possible through propensity score weighting and a natural experiment by comparing countries with differing use of treatment restrictions. We found that the use of treatment restrictions early after stroke varied widely across the European countries that participated in PRECIOUS. Although we confirmed that these restrictions were independently associated with an increased rate of death at 90 days, a comparison of death rates between countries with and those without such restrictions suggests that this relation is not causal.

**Implications of all the available evidence**

Our findings, based on analyses that minimise confounding and exploit variation in clinical practice, suggest that early treatment restrictions primarily reflect an underlying poor prognosis and cultural variation rather than increasing the risk of a premature death. Recommendations to avoid treatment restrictions in guidelines and implementation of such recommendations in care bundles or trial protocols may be premature.

## Introduction

Death of patients with acute ischaemic stroke or intracerebral haemorrhage (ICH) often occurs after a decision to withhold or withdraw life-sustaining therapies.^1^, ^2^, ^3^ In previous studies, the instalment of treatment restrictions early after acute stroke has been independently associated with an increased risk of death,^4^, ^5^, ^6^ and avoidance of such restrictions early after ICH with a greater chance of survival.^7^, ^8^, ^9^, ^10^, ^11^ These findings may have been confounded by indication, and it has remained uncertain whether the relation between early treatment restrictions and death is causal. Nevertheless, European and American guidelines on ICH suggest avoiding treatment restrictions in the first days after symptom onset and avoidance of do-not-resuscitate orders is part of care-bundle trial protocols.^12^, ^13^, ^14^

Information on the frequency of treatment restrictions early after stroke and their relations with outcomes comes from a limited number of regions and health care systems. Reported rates of early treatment restrictions range from 8.4% in a tertiary care setting in China,^15^ through 25–35% in tertiary care centres in Northern America,^9^^,^^16^ to 35–36% in tertiary care centres in Finland, the United Kingdom, and the Netherlands.^5^^,^^17^^,^^18^ In Europe, withdrawal of life-sustaining treatment in patients admitted to an intensive care unit was more frequent in northern than in southern countries,^19^ but practice variation across Europe has not been reported for patients with acute stroke.

We assessed the frequency of treatment restrictions across nine European countries in patients included in the PREvention of Complications to Improve OUtcome in elderly patients with acute Stroke (PRECIOUS) trial and used practice variation to assess whether there is any indication that the relation between early treatment restrictions and death is causal.^20^

## Methods

### Study design and participants

The current study is a post hoc analysis of the PRECIOUS trial, a European, multi-centre, 3∗2 factorial, randomised, controlled, open-label clinical trial with blinded outcome assessment (PROBE) of the preventive use of metoclopramide, ceftriaxone, paracetamol, or any combination of these, for four days in patients aged 66 years or older with acute ischaemic stroke or ICH and a score on the National Institutes of Health Stroke Scale (NIHSS) ≥ 6. Exclusion criteria included an active infection requiring antibiotic treatment, a pre-stroke score on the modified Rankin Scale (mRS) of 4 or 5, or death appearing imminent at screening. Patients were randomised within 24 h of symptom onset. PRECIOUS was conducted in academic and non-academic hospitals in Estonia, Germany, Greece, Hungary, Italy, the Netherlands, Norway, Poland, and the United Kingdom (Supplemental Figure S4). A detailed description of the study protocol, the statistical analysis plan, and the main results of the trial have previously been published and the first two have been added to the Supplemental Material.^20^, ^21^, ^22^ For this analysis, we excluded patients with a final diagnosis of a stroke mimic. The sample size was determined by the number of patients in PRECIOUS who met the eligibility criteria for the current analysis, rather than by a prospective sample size calculation.

Patients, their legal representatives or independent physicians provided written informed consent. The trial was conducted in accordance with the principles of the Declaration of Helsinki and Good Clinical Practice guidelines and was approved by the central medical ethics committee of the University Medical Center Utrecht (Reference 15–596) on 3 February 2016 and by national or local research ethics committees in all participating countries. This trial is registered with the International Standard Randomised Controlled Trial Number, ISRCTN82217627.

### Procedures

Treatment restrictions were defined as any withholding or withdrawal of life-sustaining treatment as listed below, and early treatment restrictions as those installed in the first 24 h after randomisation. Investigators prospectively recorded information on the presence of treatment restrictions at baseline and during each of the first 7 days after randomisation by selecting one or more of the following options in the study electronic case report form (eCRF) 1) none; 2) do-not-resuscitate; 3) do not intubate and ventilate; 4) withhold other treatments that may prolong life; 5) withhold food; 6) withhold fluids; 7) palliation with morphine or a benzodiazepine. Additionally, we extracted the following data from the baseline eCRF for each included patient: age; sex; ethnicity (Caucasian vs other); pre-stroke dependency (score on the mRS); stroke severity at randomisation (NIHSS sum score); stroke type (ischaemic stroke or ICH); location of stroke (left or right cerebral hemisphere, brain stem/cerebellum); country and type of including hospital (academic vs regional hospital). Finally, we extracted information on treatment allocation and death (including date) and functional outcome as assessed with the mRS at 90 days (±14 days).

### Outcomes

The primary outcome of the current study was death within 90 days (±14 days). Secondary outcomes were functional outcome defined as the score on the mRS at 90 days (±14 days), independence in activities of daily living (ADL) at 90 days defined as a score on the mRS of 2 or lower, and death within 7 days after enrolment.

### Statistical analysis

We calculated the rates of treatment restrictions in the first 24 h and in the first 7 days for each country. Differences in the proportion of patients with treatment restrictions across countries were assessed using the χ2 test. Comparisons of baseline characteristics between patients with and without restrictions were made using χ2 or Student's t-test where appropriate. For the present analysis there was no missing data for predictor and outcome variables, therefore no imputation or complete-case selection was required.

As this is an exploratory post hoc analysis no formal adjustments for multiplicity were performed.

Since causal inference from non-randomised data often relies on untestable assumptions, we estimated the possible effect of treatment restrictions on clinical outcomes via two approaches.

#### Approach 1: adjustment for confounders

First, we estimated the effect of any early treatment restriction on the primary and secondary outcomes by adjusting for known confounders (age, sex, history of diabetes mellitus, history of atrial fibrillation, pre-stroke functional dependency, baseline NIHSS, stroke type (ischaemic stroke vs ICH), time from onset of stroke to randomisation, country of enrolment, and treatment allocation in PRECIOUS) with logistic regression with inverse probability reweighting. We also performed post-hoc analyses for patients with ICH and those with ischaemic stroke separately. In addition, we visualised survival time with inverse-propensity-score reweighted Kaplan–Meier curves. Sensitivity analyses were performed for patients with a treatment restriction installed within the first seven days after randomisation. Because the assumption of no-unobserved confounding may be too strong we also investigated the sensitivity of the point estimates and the confidence intervals to unobserved confounders using E-values.^23^

#### Approach 2: comparing countries

We hypothesised that there would be country-specific differences in the rate of treatment restrictions, with some countries often installing such restrictions and other countries not or only rarely. We divided patients for this approach into two groups: patients enrolled in countries with an early treatment restriction installed in fewer than 5% of the patients (no-restriction group) and patients in countries with an early restriction installed in 5% or more of the patients (restriction group). We calculated the differences in rates of primary and secondary outcomes in both groups as estimates of the effect of treatment restrictions and adjusted the analyses with outcome regression for the same confounders as in Approach 1. We also performed post-hoc analyses of the primary outcome via this approach for patients with ICH or ischaemic stroke separately.

As s post-hoc analysis, hospital-level funnel plots with expected vs observed treatment restrictions and expected vs observed mortality were constructed based on logistic regression models including the above-mentioned prespecified baseline covariates. ‘Observed treatment restrictions’ were defined as the ratio between observed and expected restrictions, and the same applies to observed mortality.

### Role of the funding source

The study was funded by the European Union’s Horizon 2020 research and innovation programme, which had no role in study design, data collection, analysis, interpretation, writing, or publication decisions.

## Results

A total of 1493 patients from 68 sites were enrolled in PRECIOUS from April 2016 to June 2022. After excluding patients who were lost to follow-up, withdrew consent, whose informed consent form was lost or were diagnosed with a stroke mimic, 1451 patients were included in this analysis (Fig. 1). 734 patients (50.6%) were male; 1250 (86.1%) had ischaemic stroke and 201 (13.9%) ICH; their mean age was 79.7 years (SD, 7.8) and the baseline score on the NIHSS 12 (interquartile range (IQR) 8–17) (Table 1). In 950 (65.5%) patients included in this study, consent was obtained from a representative. 276 patients (19.0%) had any treatment restriction in the first 24 h from randomisation, and 360 (24.8%) in the first 7 days. Most frequently this was a do-not-resuscitate order (Fig. 2 for early restrictions, Supplementary Figure S1 for any treatment restriction). Early treatment restrictions were more frequent in patients with ICH (n = 51, 25.4%) than in patients with ischaemic stroke (n = 225, 18.0%) (Table 1). Of all patients with an early restriction, 113 (40.9%) had a do-not-resuscitate order alone, 157 (56.9%) also had an order not be intubated and ventilated, 32 (11.6%) had treatment of complications withdrawn or withheld, and in 19 (6.9%) nutrition and hydration were stopped. The rate of early treatment restrictions ranged from 0% in Estonia, Poland, and Hungary to 48.1% in the Netherlands (Table 2).

**Fig. 1:**
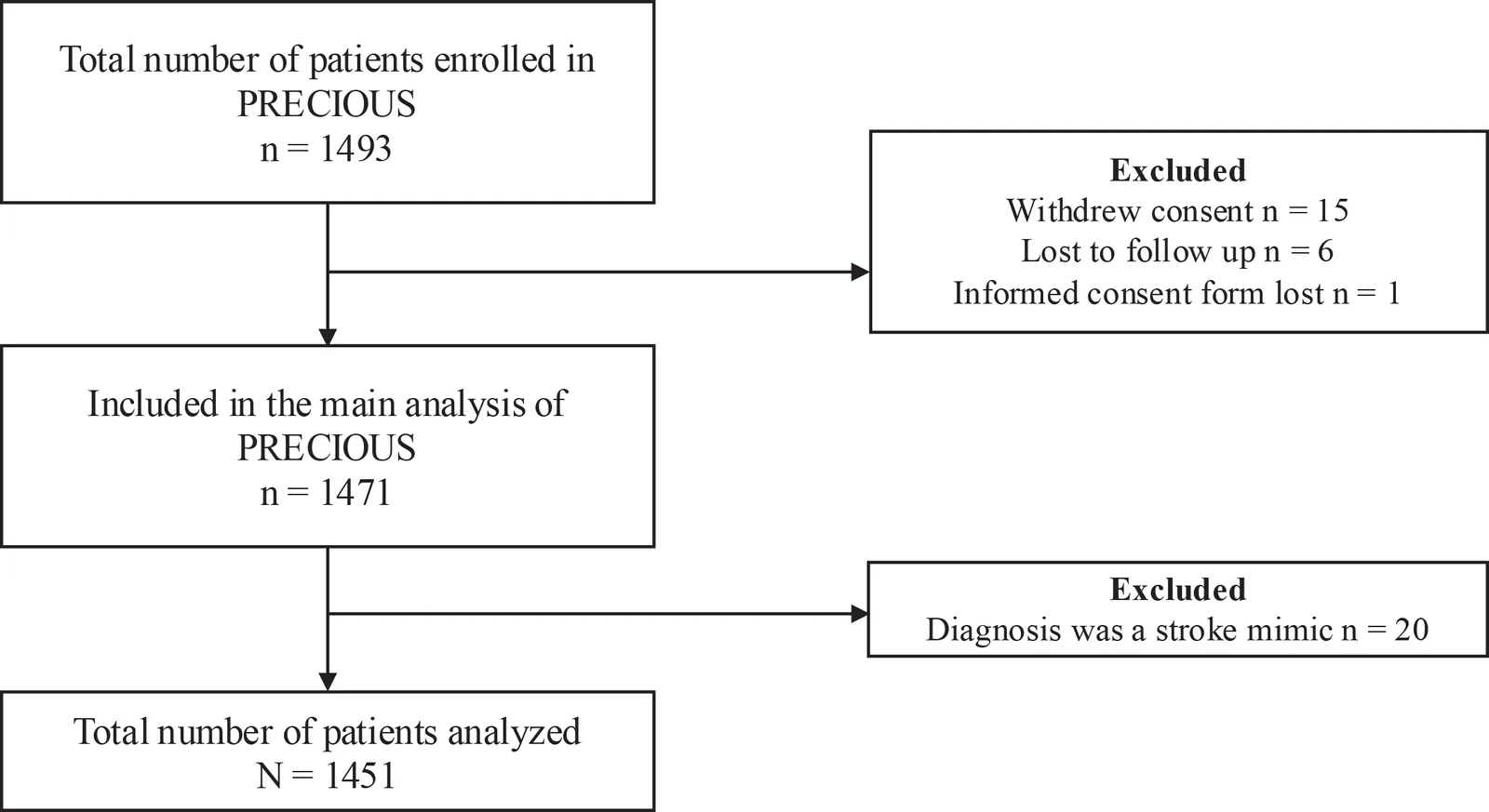
Flowchart of patient inclusion.

**Table 1:** Baseline characteristics of patients with and without early treatment restrictions.

Characteristics^a^	No early restrictions n = 1175	Early restrictions n = 276
Demographics		
Age, *years* (mean; SD)	78.9 (7.7)	83.0 (7.0)
Female sex	547 (46.6)	170 (61.6)
Ethnicity, White	1131 (96.3)	266 (96.4)
Clinical characteristics		
Intracerebral haemorrhage	150 (12.8)	51 (18.5)
Pre-stroke dependency^b^	141 (12.0)	85 (30.8)
NIHSS *score* (median; IQR)	11 (7–16)	14 (10–18)
Systolic blood pressure, *mmHg* (mean; SD)	152.4 (24.8)	153.5 (29.2)
Baseline temperature, *°C* (mean; SD)	36.4 (0.5)	36.6 (0.6)
Heart rate, *beats per minute* (mean; SD)	78.1 (17.7)	78.2 (18.6)
Treatment with IVT^c^	494 (48.1)	95 (41.9)
Treatment with EVT^c^	226 (22.0)	78 (34.4)
Onset to randomisation, *minutes* (mean; SD)	930.5 (428.0)	924.8 (472.4)
Pre-stroke feeding status		
Normal	1167 (99.3)	276 (100.0)
Oral softened foods or fluids only	8 (0.7)	0 (0.0)
Nasogastric tube	0 (0.0)	0 (0.0)
PEG	0 (0.0)	0 (0.0)
Intravenous only	0 (0.0)	0 (0.0)
Comorbidities		
Diabetes mellitus	268 (22.8)	43 (15.6)
Atrial fibrillation	326 (27.7)	103 (37.3)
Hypercholesterolaemia	427 (36.3)	92 (33.3)
Hypertension	874 (74.4)	180 (65.6)
COPD	87 (7.4)	23 (8.3)
Previous stroke	217 (18.5)	67 (24.3)
Immunocompromised	10 (0.9)	4 (1.4)
Smoking status		
Current	129 (11.1)	21 (8.0)
Ever	264 (22.8)	72 (27.5)
Never/unknown	765 (66.1)	169 (64.5)

Abbreviations: SD = standard deviation; NIHSS = National Institutes of Health Stroke Scale; IQR = interquartile range; IVT = intravenous thrombolysis; EVT = endovascular thrombectomy; PEG = percutaneous endoscopic gastrostomy; COPD = chronic obstructive pulmonary disease.

a All numbers are n (%) unless stated otherwise.

b Defined as a pre-stroke score on the modified Rankin Scale of 3–5.

c Only applicable for 1250 patients diagnosed with ischaemic stroke.

**Fig. 2:**
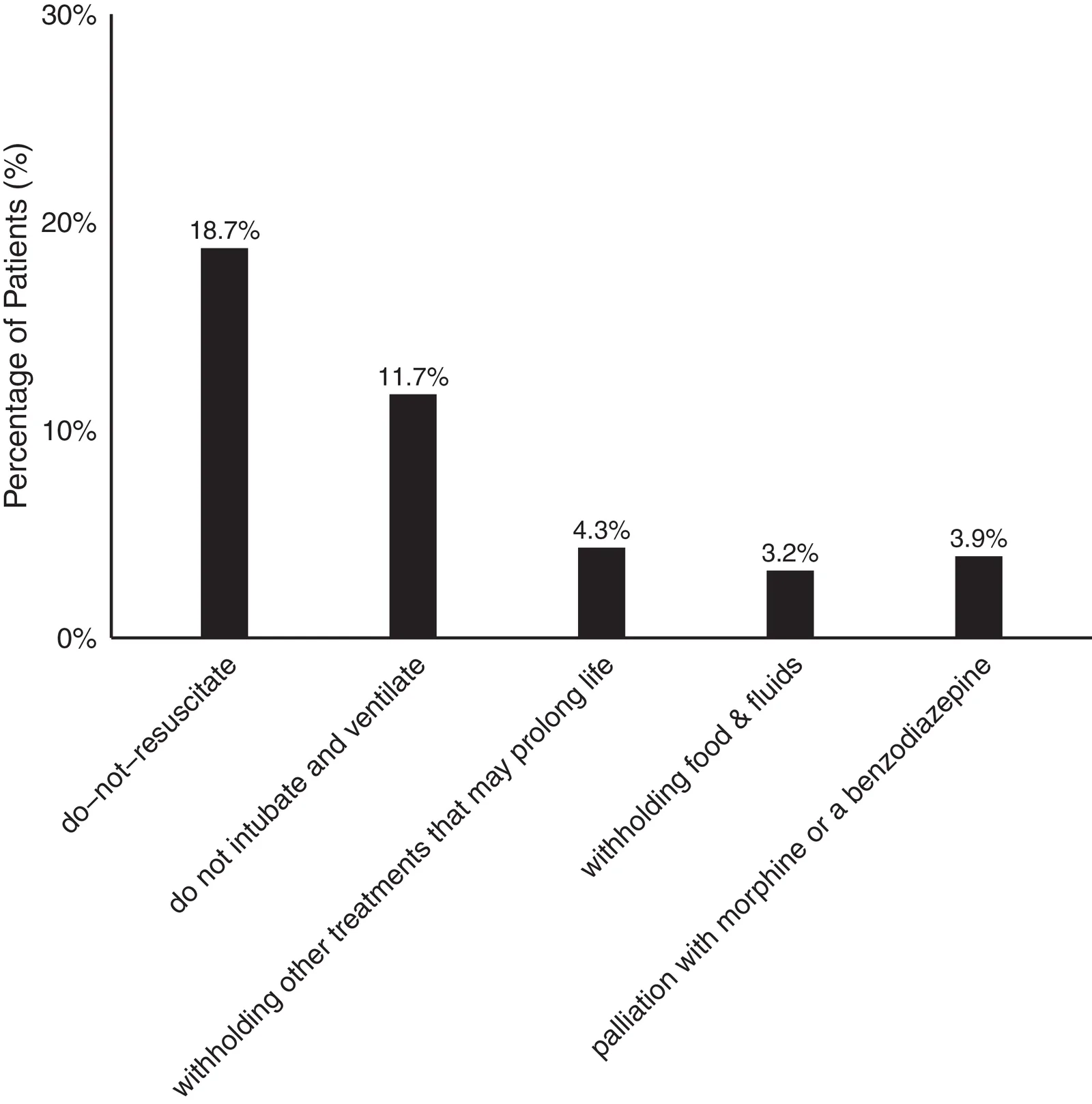
Proportions of specific types of treatment restrictions installed in the first 24 h after randomisation.

**Table 2:** Treatment restrictions per European country participating in PRECIOUS.

Country	Patients enrolled	Any treatment restriction in the first 24 h	Any treatment restriction in the first 7 days
Estonia	39	0 (0.0%)	2 (5.0%)
Germany	225	40 (17.8%)	53 (23.6%)
Greece	148	3 (2.1%)	4 (2.7%)
Hungary	52	0 (0.0%)	0 (0.0%)
Italy	224	1 (0.4%)	3 (1.3%)
Netherlands	343	165 (48.1%)	191 (55.7%)
Norway	5	1 (20.0%)	2 (40.0%)
Poland	74	0 (0.0%)	0 (0.0%)
United Kingdom	341	66 (19.4%)	105 (30.8%)

Patients with early treatment restrictions were older, more often female (but enrolled women were older than men in PRECIOUS, median age 82 vs 78 years, p < 0.001) and had greater stroke severity at baseline. Patients with treatment restrictions were more often treated with endovascular thrombectomy (EVT) than patients without treatment restrictions (Table 1).

### Approach 1

After adjustment for observed confounders, early treatment restrictions were associated with death within 90 days (39.1% vs 15.5%; inverse propensity (IP)-weighted odds ratio (OR) 3.39, 95% CI 2.71–4.25, Supplementary Figure S2), death within 7 days (16.7% vs 6.2%; IP-weighted OR 3.44, 95% CI 2.48–4.79) and with a worse functional outcome at 90 days (IP-weighted common OR 1.37 (95% CI 1.19–1.58, Table 3; Fig. 3).

**Table 3:** Primary and secondary outcomes for early restrictions, adjusted for confounders with propensity score reweighting.

Outcome^a^	No restrictions n = 1175	Early restrictions n = 276	OR+ (95% CI)
Death within 90 days	214 (18.2)	108 (39.1)	3.39 (2.71–4.25)
Death within 7 days	73 (6.2)	46 (16.7)	3.44 (2.48–4.79)
mRS at 90 days, *median (IQR)*	4 (2–5)	5 (3–6)	1.37 (1.19–1.58)
Independency in ADL^b^	358 (30.5)	32 (11.6)	0.60 (0.48–0.75)

Abbreviations: OR+ = odds ratio with inverse propensity score weighting; CI = confidence interval; mRS = modified Rankin Scale; ADL = activities of daily living.

a All numbers are n (%) unless stated otherwise.

b Defined as a score on the modified Rankin Scale of 0–2.

**Fig. 3:**
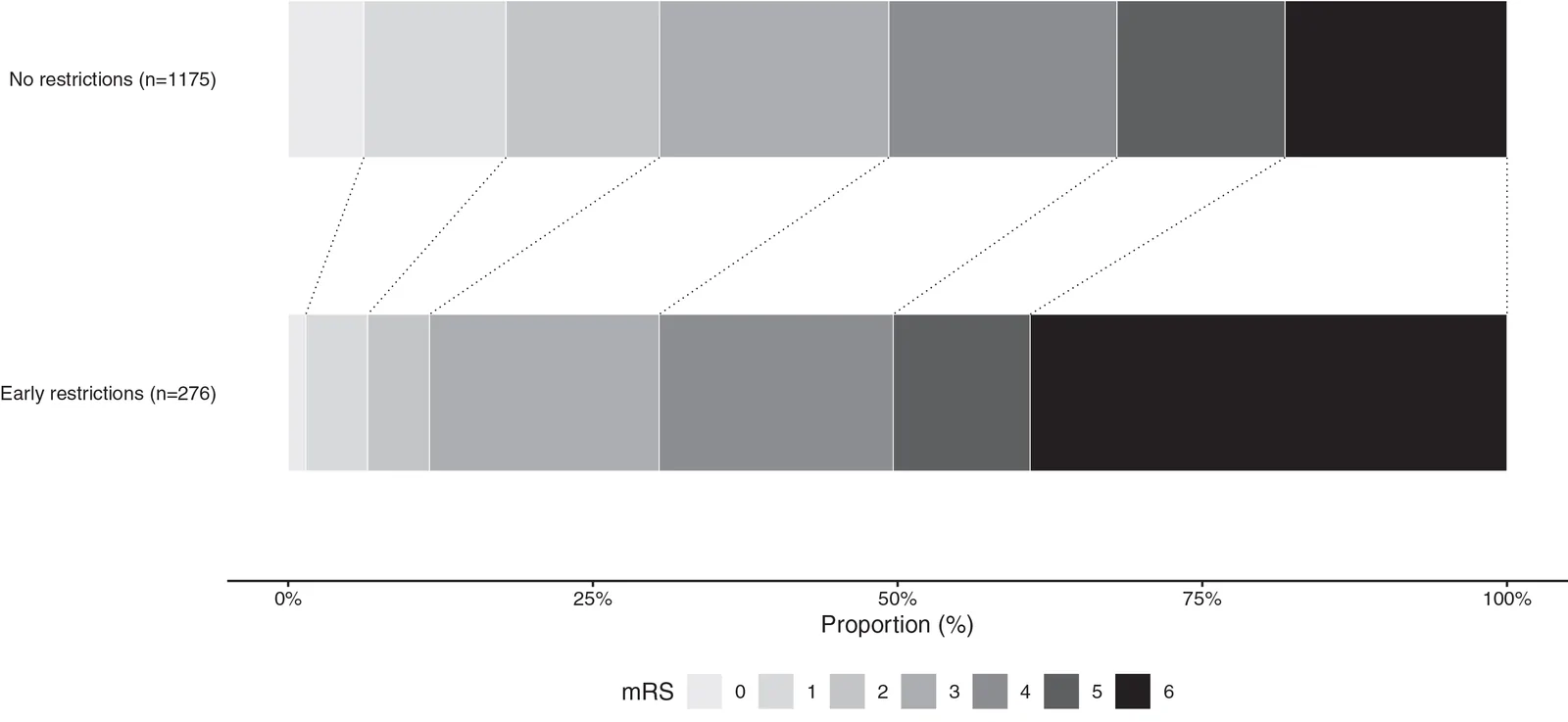
Modified Rankin Scale score at 90 days for patients with or without early treatment restrictions.

In the subgroup of patients with ICH, the corresponding IP-weighted OR for death at 90 days was 3.38 (95% CI 1.73–6.80) and for patients with ischaemic stroke 3.36 (95% CI 2.65–4.29) (Supplementary Tables S1 and S2). E-values for these analyses are presented in Supplementary Table S3 and indicate that strong unobserved confounders would need to be associated with both the exposure and outcome to explain the observed effect. Sensitivity analyses without inverse propensity weighting and for any treatment restriction separately had comparable results (Supplementary Tables S4 and S5).

### Approach 2

914 patients (63.0%) were enrolled in four countries (the Netherlands, Germany, Norway and the United Kingdom) where treatment restrictions were common and 537 (37.0%) in five countries where these were rare (Greece, Estonia, Hungary, Italy and Poland) (Table 2). Patients in the first group more often had ICH than in the second group, had more severe strokes, and had more often received reperfusion therapy (Supplementary Table S6).

Death rates were comparable between the restriction (21.3%) and no-restriction (23.5%) group (adjusted OR 1.39; 95% CI 0.81–2.39), also in subgroups of patients with ICH (adjusted OR 1.63; 95% CI 0.19–17.97) or ischaemic stroke (adjusted OR 1.32; 95% CI 0.75–2.34) (Fig. 4, Supplementary Figure S3 and Supplementary Table S7–S9). Exploratory hospital-level funnel plots demonstrated no specific pattern for the relation between expected and observed treatment restrictions but did show a funnel for the relation between expected and observed mortality (Supplementary Figures S5 and S6).

**Fig. 4:**
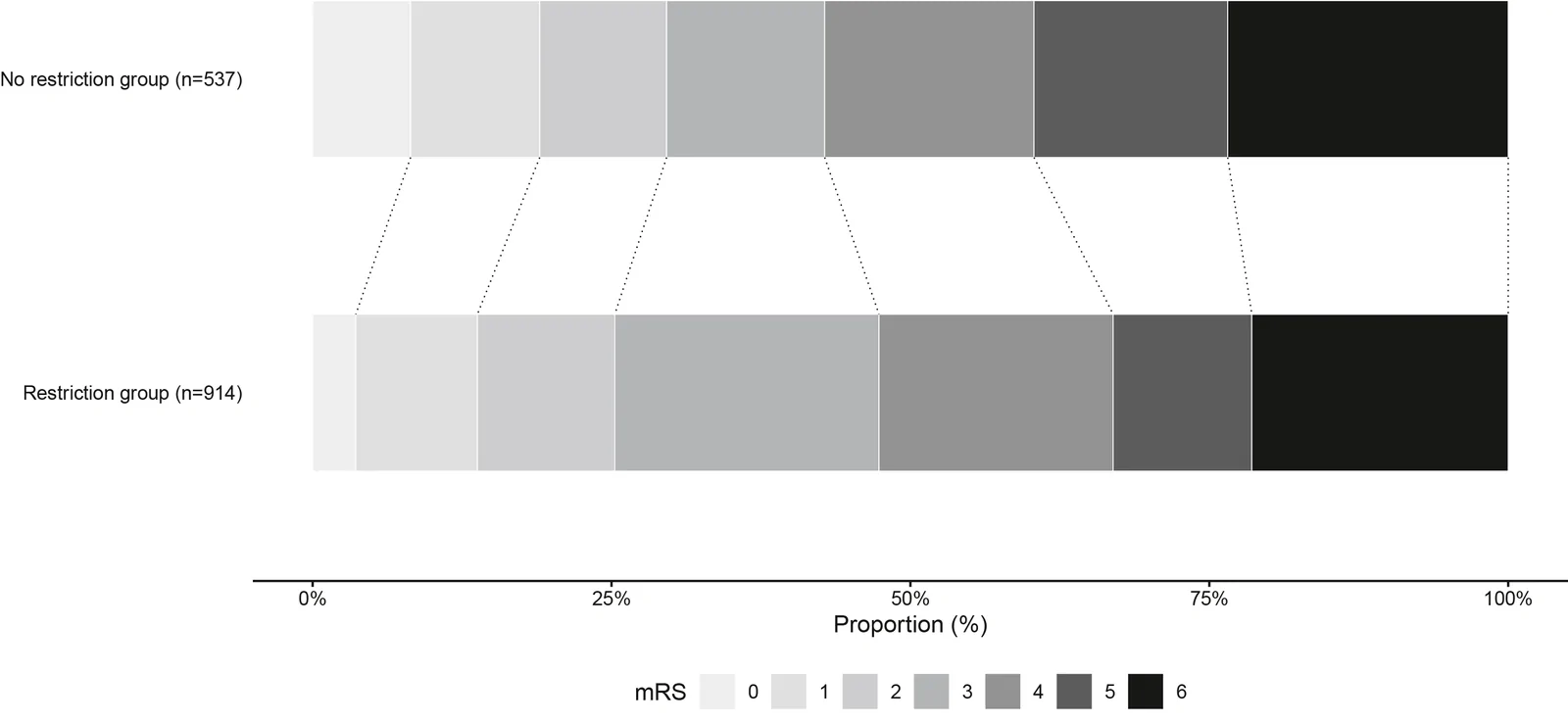
Modified Rankin Scale score at 90 days for patients in the restriction and no-restriction group based on the country of study inclusion.

## Discussion

In this European trial, about one of every five older patients with acute ischaemic stroke or ICH had a treatment restriction installed within 24 h after randomisation (or 48 h after stroke onset), but rates of treatment restrictions varied widely across the participating countries. Instalment of an early treatment restriction was independently associated with death in the first week after stroke onset, death within 90 days, and a poorer functional outcome at 90 days. However, the comparison of patients enrolled in countries where treatment restrictions are common with those in countries where these are uncommon suggests that the relation between early treatment restrictions and death at 90 days is not causal.

The prevalence of treatment restrictions in our cohort was lower than in previous observational studies in patients with ischaemic stroke or ICH in Europe, which reported restrictions installed within 24 h in 31% of the patients in one study from the Netherlands and any type of restriction at any point in time during hospital admission in one third of the patients.^5^^,^^18^ Similar percentages were seen in a Finnish study of patients with ICH.^17^ This difference is likely explained in part by differences in patient populations: our study was based on patients included in a randomised treatment trial, in which patients who had a severe pre-stroke handicap and those in whom death appeared imminent were excluded, whereas the other studies were observational and included all consecutive patients in a specific time frame. In addition, the previous observational studies were performed in the Netherlands, the United Kingdom and Finland, whereas we combined data from patients in nine European countries with some countries applying no formal treatment restrictions at all. The rate of treatment restrictions in patients included in PRECIOUS in the Netherlands was higher than reported in previous studies from the same country, possibly explained by the older age and moderately-severe to severe deficit of the patients in PRECIOUS. Patients included in Eastern or Southern European countries rarely had a treatment restriction. Reported rates of treatment restrictions in patients with ICH in our study were comparable with those in North-American studies (13–35%) and higher than in Asian (8–12%) studies.^4^^,^^11^^,^^15^^,^^16^^,^^24^, ^25^, ^26^, ^27^, ^28^, ^29^

Treatment restrictions are more likely to be installed in patients with adverse prognostic factors. Many of these adverse prognostic factors were prospectively measured in PRECIOUS and could be adjusted for, but the presence of unobserved or residual confounders is likely. Randomising patients to having a treatment restriction vs not is unethical, but the large differences in the application of treatment restrictions in PRECIOUS across countries could be seen as a somewhat ‘natural’ randomised experiment. Patients in the groups of countries with and without treatment restrictions had largely similar baseline characteristics, and known differences were adjusted for in the analyses. The absence of differences in death rates or in functional outcome between the two groups of countries, despite the large difference in the use of treatment restrictions, suggests that the observed association between treatment restrictions and poor outcome is largely driven by unobserved confounding. If early treatment restrictions would primarily prevent prolonged survival with severe disability, one might expect lower rates of survival with a score on the mRS of 5 in settings where such restrictions are more commonly implemented. We did not observe a clear pattern consistent with this hypothesis.

There are limitations to consider. First, although this was an analysis based on prospectively collected data, which improves data quality and increases the possibilities to adjust for confounding, it remains a non-randomised assessment of the exposure under question and therefore is subject to both measured and unmeasured confounding. In addition, as this was a non-prespecified post hoc analysis, the risk of bias due to multiplicity should be considered. By adding a propensity score-weighted analysis and by additionally comparing countries with and without treatment restrictions we tried to limit the effect of (un)measured confounders as much as possible. Second, apart from date of death, data were only available for the first week after stroke and for day 90. We have no information on differences in stroke care between countries after the first week, nor on the instalment of treatment restrictions past day 7. Third, as PRECIOUS was a randomised controlled trial, the observed frequency of treatment restrictions may not fully reflect routine clinical practice, as inclusion depended on trial eligibility criteria and consent procedures, leading to exclusion of patients up to the age of 65 years and those with milder strokes, and probably to underrepresentation of the most severely affected patients in whom death appeared imminent at screening. Selection bias could be further suggested by the imbalance in stroke type between the restriction and no restriction group. We have tried to adjust for this by including type of stroke in the regression analyses. Fourth, between-country comparisons are limited by potential confounding, as we lacked detailed data on differences in healthcare systems and stroke care organisation. Although by study protocol all participating countries adhered to ESO guidelines for stroke management, variations in the availability, accessibility, and quality of treatments may have influenced patient outcomes. We attempted to account for this by including country as a variable in the regression analyses; however, this approach may not fully capture relevant system-level differences. Therefore, residual confounding cannot be excluded, and between-country comparisons should be interpreted with caution. Fifth, the absence of standardised data on the cause of death during follow-up limited our ability to distinguish between potentially preventable and non-preventable deaths and to fully contextualise treatment restriction decisions. Finally, we have no information whether patients without treatment restrictions were treated more aggressively than patients with treatment restrictions. It is however likely that the differences in the rate of treatment restrictions between countries merely reflects cultural practices rather than differences in clinical care, also because stroke care in each of the participating countries was based on the same European guidelines.

### Conclusions

The rate of treatment restrictions in the acute phase of stroke differs greatly across Europe. Such restrictions are associated with a poor functional outcome or death, but this is likely driven by unmeasured confounding. Restrictions likely act as marker for poor prognosis, and we found no strong indication that the relation with outcome is causal. Therefore, recommendations in guidelines to avoid early treatment restrictions and inclusion of such recommendations in care bundles appear premature.

## Contributors

WMS, JCdJ, HR and HBvdW conceptualised the study and had full access to all the data. WMS, JCdJ, WVA and HR curated the data. WMS and WVA performed the formal statistical analyses. WMS and HBvdW wrote the first draft of the manuscript. WMS, WVA, JCdJ, HR, GN, JK, AHA, GT, LC, AC, IK-J, MRM, PMB and HBvdW all contributed to review and editing of the manuscript and approved the final version for submission. The PRECIOUS investigators coordinated the trial at the local study sites.

## Data sharing statement

Anonymised individual participant data that underlie the results reported in this article are available from the corresponding author upon reasonable request, subject to privacy and other relevant regulation.

## Declaration of interests

WMS, JCdJ, HR, GN, JK, AHA, GT, LC, AC, IK-J, MRM, PMB and HBvdW all report grants from the European Union, all paid to their institutions.

HBvdW reports having received grants from the Dutch Heart Foundation and Stryker for research, and funding for consultancy from Bayer, Boehringer Ingelheim, and TargED, all paid to his institution.

PMB is Stroke Association Professor Stroke Medicine and an emeritus NIHR Senior Investigator; he has stock options in CoMind and DiaMedica and receives devices from Phagenesis for the NIHR-funded PhEAST trial.

## Appendix A Supplementary data

PRECIOUS Trial Study Protocol

PRECIOUS Trial Statistical Analysis Plan

PRECIOUS Treatment Restrictions Protocol
