# Supplementary material for: Treatment restrictions after acute stroke across nine European countries and their association with 90-day mortality: a post hoc analysis of the PRECIOUS randomised controlled trial
Source: Lancet Reg Health Eur. 2026 Oct 14;71:101894. doi: 10.1016/j.lanepe.2026.101894 (PMC13694901; doi:10.1016/j.lanepe.2026.101894)
Supplement: Supplementary file 3 [file mmc3.docx]

**Treatment restrictions after stroke: variation across Europe and association with outcome – protocol for a PRECIOUS substudy**

**Version 1.1; 23 January 2026**

**Protocol amendment**

23-01-2026: A latent decision threshold model that was included as a third statistical approach was omitted from the final protocol because preliminary evaluation indicated that it produced unstable/non-interpretable estimates

**1. BACKGROUND AND RATIONALE**

Of all patients with stroke, 20-35% die within one month.^1-3^ Most deaths occur after a decision to withhold or withdraw life-sustaining therapies, often after a complication.^4-6^ Early instalment of such treatment restrictions in patients with stroke has been associated with a higher risk of death,^7-9^ and avoidance of treatment restrictions in the first days after intracranial haemorrhage with a lower 30-day mortality.^10-11^ Treatment restrictions are often installed in the acute phase after stroke and the patient him/herself is often not involved because of a diminished capacity to make such decisions.^12^ The frequency of treatment restrictions varies between stroke diagnosis and country of the patients. In 35-51% of patients with intracerebral haemorrhage treatment restrictions are installed in Europe,^8,13^ 25-35% in Northern America,^7,14^ and 8.4% in China.^15^ Possibly, these restrictions vary due to cultural or religious differences,^16^ but direct comparison between countries is complicated by differences in study population. In addition, studies on treatment restrictions are often limited by their retrospective nature, and the timing of the instalment of treatment restrictions is most often not reported.

In the PREvention of Complications to Improve OUtcome in elderly patients with acute Stroke (PRECIOUS) trial, a multicentre randomized trial in elderly patients with stroke in nine countries in Europe, information about treatment restrictions in the first seven days after stroke was systematically and prospectively collected.^17^ We aim to describe the frequency of treatment restrictions in patients with stroke in the participating countries and assess the association of treatment restrictions with death and functional outcome at 7 and 90 days.

**2. STUDY DESIGN**

*Study Objectives*

Primary aim:

1. To estimate the effect of treatment restrictions installed in the first 24 hours after randomization on mortality within 90 days.

Secondary aims:

2. To describe the variation, timing and types of treatment restrictions installed in the first week after stroke across European countries that participated in PRECIOUS.

3. To estimate the effect of treatment restrictions installed in the first 24 hours days after stroke randomization on mortality within 7 days.

4. To estimate the effect of treatment restrictions installed in the first 24 hours after randomization on functional outcome measured with the modified Rankin Scale (mRS) at 90 days.

5. To estimate the effect of treatment restrictions installed in the first 24 hours after randomization on survival time.

*Study population*

PRECIOUS was an international, multi-centre, 3*2-factorial, randomised, controlled, open-label clinical trial with blinded outcome assessment (PROBE) of the preventive use of metoclopramide, ceftriaxone, paracetamol, or any combination of these, for four days in patients aged 66 years or older with acute ischaemic stroke or intracerebral haemorrhage and an score on the National Institutes of Health Stroke Scale (NIHSS) ≥ 6. Exclusion criteria included an active infection requiring antibiotic treatment, a pre-stroke score on the mRS of ≥ 4 or death appearing imminent at screening. A detailed description of the study protocol, the statistical analysis plan, and the main results have previously been published.^18-20^ For this analysis we will exclude patients diagnosed with a stroke mimic.

*Outcomes*

The primary outcome of the current study will be death within 90 days. Secondary outcomes will be functional outcome defined as the score on the mRS at 90 days, death or dependency at 90 days defined as score on the mRS of 3 or higher, death within 7 days and the proportion of treatment restrictions installed for the full study population and per country.

*Data collection*

Investigators in PRECIOUS were asked to provide information in the study electronic case report file (eCRF) about the presence of treatment restrictions at baseline and during each of the first 7 days after randomization by selecting one or more of the following options: 1) none; 2) do-not-resuscitate; 3) do not ventilate and intubate; 4) withholding other treatments that may prolong life; 5) withholding food; 6) withholding fluids; 7) Palliation with morphine or a benzodiazepine; 8) not applicable. Additionally, we will extract the following data from the eCRF for each included patient: age; sex; ethnicity (Caucasian versus other); pre-stroke disability (score on the mRS); stroke severity at randomisation (NIHSS sum score); stroke type (ischaemic stroke or intracerebral haemorrhage (ICH); location of stroke (left cerebral hemisphere, right cerebral hemisphere, brain stem/cerebellum); country and type of including hospital (academic versus regional hospital), allocation of study treatment, and functional outcome (including death) as assessed with the mRS at 90 days (+-14 days); and date of death.

*Identification of causal effect – statistical analysis plan*

The primary aim of this study is to estimate the causal effect of installing treatment restrictions on mortality at 90 days and secondarily on death within 7 days and functional outcome based on the score on the mRS at 90 days. Since instalment of treatment restrictions was not a randomized intervention we need to rely on assumptions for estimating this effect. We will rely on several independent assumptions when analysing the data to increase the robustness of our estimates. The assumptions are: 1. the comparability of populations and outcomes across countries, 2. the absence or at least scarcity of unobserved confounders and 3. the correctness of a latent threshold model for deciding on treatment restrictions. We now briefly describe how these assumptions lead to different estimation approaches including a plan for the statistical analysis, allowing us to investigate the effect of interest.

*Approach 1: adjustment for confounders*

First, we can estimate the effect of treatment restrictions on survival and functional outcomes with outcome regression to adjust for known confounders with and without inverse-propensity score reweighting. This lies under the assumption there are no or at least few unobserved confounders. For this analysis we will compare patients with treatment restrictions installed within 24 hours after randomization with patients who had no treatment restrictions within 24 hours. We chose for treatment restrictions within 24 hours because patients in whom death was imminent were not eligible for inclusion in PRECIOUS. It is therefore unlikely that these treatment restrictions were installed because a clinician thought that it was inevitable that the patient would die on short notice, as could be the case for treatment restrictions later in the first week.

Differences between groups will be assessed with chi-squared testing for categorical variables, two-sample t-test for continuous normally distributed data and Mann-Whitney U for non-normally distributed data. To adjust for known confounders (age, sex, medical history of diabetes, pre-stroke functional dependency, baseline NIHSS and type of stroke as well as whether patients were randomized to a treatment stratum in PRECIOUS) for the primary and secondary outcome we will perform multivariable logistic regression for death at 90 days and death at 7 days, and multivariable ordinal regression for the mRS at 90 days. Due to the many parameters involved in the categorical distribution of mRS scores, we only apply inverse-propensity score reweighting for that outcome definition. Furthermore we will visualize survival time with a Kaplan-Meier curve for patients with and without treatment restrictions. Sensitivity analyses will be performed for patients with a treatment restriction installed within the first seven days after randomization.

*Approach 2: observed outcomes in no-restriction countries*

We hypothesize there will be country-specific differences in treatment restrictions installed in the first week after stroke, with countries often installing treatment restrictions and countries not or very rarely installing these. As all patients were treated in European countries abiding the European Stroke Organisation guidelines on acute stroke treatment,^21,22^ we assume that all patients were treated equally. We will divide patients for this approach into two groups, patients enrolled in countries with less than 5% of patients with a treatment restriction installed within 24 hours after randomization (no-restriction group) and patients in countries with more than 5% of patients with a treatment restriction installed within 24 hours after randomization (restriction group). It may be argued that the expected outcomes of a patient *if no restriction would be applied* would be the same in the ‘no-restriction’ group as in the restriction group, and the same for *if a restriction would be applied.* This is formally known as exchangeability of potential outcomes.^23^ Under this assumption, we will calculate the average rate of death and the distribution of mRS outcomes in the no-restriction group and calculate difference in outcomes with the ‘restriction’ group as our estimate of the effect.

But as although eligibility criteria for patients to be randomized were identical for all countries this does not guarantee all baseline characteristics were equally distributed over these countries. We will therefore also make comparisons between these two groups with chi-squared testing for categorical variables, two-sample t-test for continuous normally distributed data and Mann-Whitney U for non-normally distributed data. To adjust for known confounders for the primary and secondary outcomes we will adjust for (1) pre-specified confounders: age, sex, medical history of diabetes, pre-stroke functional dependency, baseline NIHSS and type of stroke as well as (2) whether patients were randomized to a treatment stratum in PRECIOUS. We will perform multivariable logistic regression for death at 90 days and death at 7 days, and multivariable ordinal regression for the mRS at 90 days. In addition we will visualize survival with a Kaplan-Meier curve for restriction countries and no-restriction countries. In addition we will perform sensitivity analyses for groups divided by countries with and without treatment restriction installed within the first week after randomization.

**REFERENCES**

1. Lozano R, Naghavi M, Foreman K et al. Global and regional mortality from 235 causes of

death for 20 age groups in 1990 and 2010: a systematic analysis for the Global Burden of

Disease Study 2010. Lancet 2012;380(9859):2095-2128

2. Feigin VL, Lawes CM, Bennett DA, Barker-Collo SL, Parag V. Worldwide stroke incidence and early case fatality reported in 56 population-based studies: a systematic review. Lancet Neurol. 2009;8(4):355-369.

3. Heuschmann PU, Wiedmann S, Wellwood I et al. Three-month stroke outcome: the European Registers of Stroke (EROS) investigators. Neurology. 2011;76(2):159-165.

4. Kelly AG, Hoskins KD, Holloway RG. Early stroke mortality, patient preferences, and the withdrawal of care bias. Neurology 2012;

5. Becker KJ, Baxter AB, Cohen WA, et al. Withdrawal of support in intracerebral hemorrhage may lead to self-fulfilling prophecies.

6. Sluis WM, Hinsenveld WH, Goldhoorn RB, Potters LH, Bruggeman AA, van der Hoorn A, Bot JC, van Oostenbrugge RJ, Lingsma HF, Hofmeijer J, van Zwam WH, Blm Majoie C, Bart van der Worp H. Timing and causes of death after endovascular thrombectomy in patients with acute ischemic stroke. Eur Stroke J. 2023 Mar;8(1):215-223.

7. Zahuranec DB, Brown DL, Lisabeth LD, Gonzales NR, Longwell PJ, Smith MA, et al. Early care limitations independently predict mortality after intracerebral hemorrhage. Neurology. 2007;68:1651-1657.

8. Reinink H, Konya B, Geurts M, Kappelle LJ, van der Worp HB. Treatment Restrictions and the Risk of Death in Patients With Ischemic Stroke or Intracerebral Hemorrhage. Stroke. 2020;51(9):2683-2689.

9. Geurts M, de Kort FA, de Kort PL, et al. Treatment restrictions in patients with severe stroke are associated with an increased risk of death. Eur Stroke J. 2017;2(3):244-249.

10. Creutzfeldt CJ, Becker KJ, Weinstein JR, Khot SP, McPharlin TO, Ton TG, Longstreth WT, Tirschwell DL. Do-not-attempt-resuscitation orders and prognostic models for intraparenchymal hemorrhage.Crit Care Med. 2011; 39:158–162.

11. Zahuranec DB, Morgenstern LB, Sánchez BN, Resnicow K, White DB, Hemphill JCDo-not-resuscitate orders and predictive models after intracerebral hemorrhage.Neurology. 2010; 75:626–633.

12. Thomalla G, Boutitie F, Fiebach JB et al. Effect of Informed Consent on Patient Characteristics in a Stroke Thrombolysis Trial. Neurology. 2017 Sep 26;89(13):1400-1407

13. Silvennoinen K, Meretoja A, Strbian D, Putaala J, Kaste M, Tatlisumak T. Do-not-resuscitate (DNR) orders in patients with intracerebral hemorrhage.Int J Stroke. 2014; 9:53–58.

14. Hemphill JC, Newman J, Zhao S, Johnston SC. Hospital usage of early do-not-resuscitate orders and outcome after intracerebral hemorrhage.Stroke. 2004; 35:1130–1134.

15. Yang TC, Li JG, Guo W. Do not resuscitate orders for patients with intracerebral hemorrhage: experience from a Chinese tertiary care center.Eur Neurol. 2015; 73:144–149.

16. Sprung, C.L., Maia, P., Bulow, HH. et al. The importance of religious affiliation and culture on end-of-life decisions in European intensive care units. Intensive Care Med 33, 1732–1739 (2007)

17. de Jonge JC, Sluis WM, Reinink H, Bath PM, Woodhouse LJ, Zweedijk B, van de Beek D, Aamodt AH, Alpers I, Ciccone A, Csiba L, Demotes J, Kõrv J, Kurkowska-Jastrzebska I, Dawson J, Macleod MR, Ntaios G, Poli S, Milionis H, Ricci S, Cenciarelli S, Candelaresi P, de Bruijn SF, Pathansali R, Krishnan K, Clarke B, Thomalla G, van der Worp HB; PRECIOUS investigators. Prevention of infections and fever to improve outcome in older patients with acute stroke (PRECIOUS): a randomised, open, phase III, multifactorial, clinical trial with blinded outcome assessment. Lancet Reg Health Eur. 2023 Dec 1;36:100782

18. Reinink H, de Jonge JC, Bath PM, van de Beek D, Berge E, Borregaard S, Ciccone A, Csiba L, Demotes J, Dippel DW, Kõrv J, Kurkowska-Jastrzebska I, Lees KR, Macleod MR, Ntaios G, Randall G, Thomalla G, van der Worp HB.PRECIOUS: PREvention of Complications to Improve OUtcome in elderly patients with acute Stroke. Rationale and design of a randomised, open, phase III, clinical trial with blinded outcome assessment.

Eur Stroke J. 2018 Sep;3(3):291-298.

19. de Jonge JC, Woodhouse LJ, Reinink H, van der Worp HB, Bath PM; PRECIOUS investigators. PRECIOUS: PREvention of Complications to Improve OUtcome in elderly patients with acute Stroke-statistical analysis plan of a randomised, open, phase III, clinical trial with blinded outcome assessment. Trials. 2020;21(1):884.

20. de Jonge JC, Sluis WM, Reinink H, Bath PM, Woodhouse LJ, Zweedijk B, van de Beek D, Aamodt AH, Alpers I, Ciccone A, Csiba L, Demotes J, Kõrv J, Kurkowska-Jastrzebska I, Dawson J, Macleod MR, Ntaios G, Poli S, Milionis H, Ricci S, Cenciarelli S, Candelaresi P, de Bruijn SF, Pathansali R, Krishnan K, Clarke B, Thomalla G, van der Worp HB; PRECIOUS investigators. Prevention of infections and fever to improve outcome in older patients with acute stroke (PRECIOUS): a randomised, open, phase III, multifactorial, clinical trial with blinded outcome assessment. Lancet Reg Health Eur. 2023 Dec 1;36:100782

21. Steiner T, Al-Shahi Salman R, Beer R, Christensen H, Cordonnier C, Csiba L, Forsting M, Harnof S, Klijn CJ, Krieger D, Mendelow AD, Molina C, Montaner J, Overgaard K, Petersson J, Roine RO, Schmutzhard E, Schwerdtfeger K, Stapf C, Tatlisumak T, Thomas BM, Toni D, Unterberg A, Wagner M; European Stroke Organisation. European Stroke Organisation (ESO) guidelines for the management of spontaneous intracerebral hemorrhage. Int J Stroke. 2014 Oct;9(7):840-55

22. Berge E, Whiteley W, Audebert H, De Marchis GM, Fonseca AC, Padiglioni C, de la Ossa NP, Strbian D, Tsivgoulis G, Turc G. European Stroke Organisation (ESO) guidelines on intravenous thrombolysis for acute ischaemic stroke. Eur Stroke J. 2021 Mar;6(1):I-LXII.

23. Hernán MA, Robins JM. What if?: Chapter 2. Boca Raton, FL: Chapman & Hall/CRC; 2020
