# Supplementary material for: Treatment restrictions after acute stroke across nine European countries and their association with 90-day mortality: a post hoc analysis of the PRECIOUS randomised controlled trial
Source: Lancet Reg Health Eur. 2026 Oct 14;71:101894. doi: 10.1016/j.lanepe.2026.101894 (PMC13694901; doi:10.1016/j.lanepe.2026.101894)
Supplement: Supplementary file 4 [file mmc4.docx]

**Supplementary Figure S1.** Proportions of specific types of treatment restrictions installed within the first seven days after randomisation.

**Supplementary Figure S2.** Kaplan-Meier curve for 90-day survival for patients with early restrictions and patients without restriction early restrictions, adjusted for confounders with inverse propensity score reweighting

**Supplementary Table S1**. Primary and secondary outcomes for early restrictions in a subgroup of patients with intracerebral haemorrhage, adjusted for confounders with propensity score reweighting

| **Outcome*** | **No restrictions**  n = 150 | **Early restrictions**  n = 51 | **OR+ (95% CI)** |
| --- | --- | --- | --- |
| Death within 90 days  Death within 7 days  mRS at 90 days, *median (IQR)*  Independency in ADL† | 24 (16·0)  1 (5·3)  5 (4-6)  27 (18·0) | 18 (35·3)  9 (17·6)  5 (5-7)  5 (9·8) | 3·38 (1·73 – 6·80)  7·56 (2·75 – 24·52)  1·49 (0·97 – 2·29)  0·71 (0·31 – 1·62) |

**Abbreviations**: OR+ = odds ratio with inverse propensity score weighting; CI = confidence interval; mRS = modified Rankin Scale; ADL = activities of daily living

* all numbers are n (%) unless stated otherwise

† defined as a score on the mRS of 0-2

**Supplementary Table S2**. Primary and secondary outcomes for early restrictions in a subgroup of patients with ischaemic stroke, adjusted for confounders with propensity score reweighting

| **Outcome*** | **No restrictions**  n = 1025 | **Early restrictions**  n = 225 | **OR+ (95% CI)** |
| --- | --- | --- | --- |
| Death within 90 days  Death within 7 days  mRS at 90 days, *median (IQR)*  Independency in ADL† | 190 (18·5)  65 (6·3)  4 (3-6)  331 (32·3) | 90 (40·0)  37 (16·4)  6 (4-7)  27 (12·0) | 3·36 (2·65 – 4·29)  3·22 (2·29 – 4·60)  1·36 (1·17 – 1·58)  0·60 (0·47 – 0·76) |

**Abbreviations**: OR+ = odds ratio with inverse propensity score weighting; CI = confidence interval; mRS = modified Rankin Scale; ADL = activities of daily living

* all numbers are n (%) unless stated otherwise

† defined as a score on the mRS of 0-2

**Supplementary Table S3**. E-values for point estimates for the primary and secondary outcomes

| **Outcome** | **E-value** |
| --- | --- |
| Death within 90 days  Death within 7 days  mRS at 90 days  Independence in ADL† | 3·09  3·11  1·61  1·90 |

**Abbreviations**: OR+ = odds ratio with inverse propensity score reweighting; CI = confidence interval; mRS = modified Rankin scale; ADL = activities of daily living

† defined as a score on the mRS of 0-2

**Supplementary Table S4.** Primary and secondary outcomes for early restrictions, adjusted for confounders without propensity score weighting

| **Outcome*** | **No restrictions**  n = 1175 | **Early restrictions**  n = 276 | **OR (95% CI)** |
| --- | --- | --- | --- |
| Death within 90 days  Death within 7 days  mRS at 90 days,  *median (IQR)*  Independency in ADL† | 214 (18·2)  73 (6·2)  4 (2-5)  358 (30·5) | 108 (39·1)  46 (16·7)  5 (3-6)  32 (11·6) | 2·27 (1·27 – 4·07)  2·46 (1·09 – 5·54)  1·35 (0·89 – 2·05)  0·64 (0·31 – 1·32) |

**Abbreviations**: OR = odds ratio; CI = confidence interval; mRS = modified Rankin scale; ADL = activities of daily living

* all numbers are n (%) unless stated otherwise

† defined as a score on the mRS of 0-2

**Supplementary Table S5**. Primary and secondary outcomes for any restriction, adjusted for confounders with propensity score reweighting

| **Outcome** | **No restrictions**  n = 1091 | **Treatment restrictions**  n = 360 | **OR+ (95% CI)** |
| --- | --- | --- | --- |
| Death within 90 days  Death within 7 days  mRS at 90 days, *median (IQR)*  Independence in ADL† | 169 (15·5)  47 (4·3)  3 (2 – 5)  355 (32·5) | 153 (42·5)  72 (20·0)  5 (3 – 6)  35 (9·7) | 5·30 (4·20 – 6·74)  8·56 (6·01 – 12·40)  2·49 (2·13 – 2·89)  0·47 (0·38 – 0·60) |

**Abbreviations**: OR+ = odds ratio with inverse propensity score weighting; CI = confidence interval; mRS = modified Rankin scale; ADL = activities of daily living

* all numbers are n (%) unless stated otherwise

† defined as a score on the mRS of 0-2

**Supplementary Table S6**. Baseline characteristics of patients in the restriction and no-restriction group based on the country of study inclusion

| **Characteristics*** | **No restriction-group**  n = 537 | **Restriction-group**  n = 914 |
| --- | --- | --- |
| **Demographics**  Age, *years* (mean; SD)  Female sex  Ethnicity, White | 80·1 (8·3)  277 (51·6)  537 (100·) | 79·4 (7·5)  440 (48·1)  860 (94·1) |
| **Clinical characteristics**  Intracerebral haemorrhage  Pre-stroke dependency ^†^  NIHSS *score* (median; IQR)  Systolic blood pressure, *mmHg* (mean; SD)  Baseline temperature, *°C* (mean; SD)  Heart rate, *beats per minute* (mean; SD)  Treatment with IVT^‡^  Treatment with EVT^‡^  Onset to randomisation, *minutes* (mean; SD)  Pre-stroke feeding status   - *Normal* - *Oral softened foods or fluids only* - *Nasogastric tube* - *PEG* - *Intravenous only* | 53 (9·9)  65 (12·1)  10 (7-16)  150·1 (23·3)  36·4 (0·4)  77·3 (15·8)  207 (42·8)  63 (13·0)  898·3 (447·4)  533 (99·3)  4 (0·7)  0 (0·0)  0 (0·0)  0 (0·0) | 148 (16·2)  161 (17·6)  12 (8-17)  154·0 (26·8)  36·5 (0·6)  78·5 (18·9)  382 (49·7)  241 (31·3)  950·6 (427·6)  910 (99·6)  4 (0·4)  0 (0·0)  0 (0·0)  0 (0·0) |
| **Comorbidities**  Diabetes Mellitus  Atrial fibrillation  Hypercholesterolemia  Hypertension  COPD  Previous stroke  Immunocompromised  Smoking status   - *Current* - *Ever* - *Never/unknown* | 149 (27·7)  143 (26·6)  224 (41·7)  449 (83·6)  51 (9·5)  102 (19·0)  7 (1·3)  68 (12·7)  82 (15·4)  384 (71·9) | 162 (17·7)  286 (31·3)  295 (32·3)  605 (66·2)  59 (6·5)  182 (19·9)  7 (0·8)  82 (9·3)  254 (28·7)  550 (62·1) |

**Abbreviations**: SD = standard deviation; NIHSS = National Institute of Health Stroke Scale; IQR = interquartile range; IVT = intravenous thrombolysis; EVT = endovascular thrombectomy; PEG = percutaneous endoscopic gastrostomy; COPD = chronic obstructive pulmonary disease

* all numbers are n(%) unless stated otherwise

† defined as a pre-stroke score on the modified Rankin Scale of 0 – 2

‡ only applicable for 1250 patients diagnosed with ischaemic stroke

**Supplementary Table S7**. Primary and secondary outcomes in the restrictions versus no restriction groups based on the country of study inclusion, adjusted for confounders

| **Outcome*** | **No restriction group**  n = 537 | **Restriction group**  n = 914 | **OR+ (95% CI)** |
| --- | --- | --- | --- |
| Death within 90 days  Death within 7 days  mRS at 90 days, *median (IQR)*  Independence in ADL† | 196 (23·5)  43 (8·0)  4 (2-5)  159 (29·6) | 195 (21·3)  76 (8·3)  4 (2-5)  231 (25·3) | 1·39 (0·81 – 2·39)  1·36 (0·64 – 2·98)  0·84 (0·60 – 1·18)  1·22 (0·69 – 2·16) |

**Abbreviations**: OR+ = odds ratio with inverse propensity score weighting; CI = confidence interval; mRS = modified Rankin Scale; ADL = activities of daily living

* all numbers are n (%) unless stated otherwise

† defined as a score on the mRS of 0 – 2.

**Supplementary Figure S3**. Kaplan-Meier curve for 90-day survival for patients in the restriction and no restriction groups based on the country of inclusion, adjusted for confounders with inverse propensity score reweighting.

**Supplementary Table S8**. Primary and secondary outcomes in patients with intracerebral haemorrhage enrolled in the restrictions versus no restriction group based on the country of study inclusion, adjusted for confounders

| **Outcome*** | **No restriction group**  n = 53 | **Restriction group**  n = 148 | **OR+ (95% CI)** |
| --- | --- | --- | --- |
| Death within 90 days  Death within 7 days  mRS at 90 days, *median (IQR)*  Independence in ADL† | 12 (22·6)  6 (11·3)  5 (4 – 6)  9 (17·0) | 30 (20·3)  11 (7·4)  5 (4 – 6)  23 (15·5) | 1·63 (0·19 – 17·97)  6·29 (0·02 – 16533·93)  0·40 (0·11 – 1·37)  18·61 (0·18 – 14538·84) |

**Abbreviations**: OR+ = odds ratio with inverse propensity score weighting; CI = confidence interval; mRS = modified Rankin Scale; ADL = activities of daily living

* all numbers are n (%) unless stated otherwise

† defined as a score on the mRS of 0-2

**Supplementary Table S9**. Primary and secondary outcomes in patients with ischaemic stroke enrolled in the restrictions versus no restriction group based on the country of study inclusion, adjusted for confounders

| **Outcome*** | **No restriction group**  n = 484 | **Restriction group**  n = 766 | **OR+ (95% CI)** |
| --- | --- | --- | --- |
| Death within 90 days  Death within 7 days  mRS at 90 days, *median (IQR)*  Independence in ADL† | 114 (23·6)  37 (7·6)  5 (3 – 6)  150 (31·0) | 166 (21·7)  65 (8·5)  4 (3 – 6)  208 (27·2) | 1·32 (0·75 – 2·34)  1·36 (0·61 – 3·13)  0·89 (0·62 – 1·26)  1·14 (0·63 – 2·06) |

**Abbreviations**: OR+ = odds ratio with inverse propensity score weighting; CI = confidence interval; mRS = modified Rankin Scale; ADL = activities of daily living

* all numbers are n (%) unless stated otherwise

† defined as a score on the mRS of 0-2

**Supplementary Figure S4.** All participating countries in PRECIOUS

**Supplementary Figure S5.** Hospital-level funnel plot of expected versus observed treatment restrictions

**Supplementary Figure S6.** Hospital-level funnel plot of expected versus observed deaths
